# 

**Published:** 1902-08

**Authors:** 


*Fifty-Eighth Year.*

VOL. LVIII.

WHOLE NUMBER,

DCLXIX.

AUGUST, 1902.

New Series.
VOL. XLII.
No. 1.

### BUFFALO MEDICAL JOURNAL

**Established 1845 by AUSTIN FLINT, M. D.**

**EDITOR:**

WILLIAM WARREN POTTER, M. D.

**ASSISTANT EDITORS:**

WILLIAM C. KRAUSS, M. D. NELSON W. WILSON, M. D.

**ASSOCIATE EDITORS:**

JAMES WRIGHT PUTNAM, M. D. ERNEST WENDE, M. D.
JOHN PARMENTER, M. D. JOHN A. MILLER, PH. D
HARVEY R. GAYLORD, M. D. MAUD J. FRYE, M. D.

SCRIPTA FERUNT ANNOS

LIBRARY
SURGEON GENERAL'S OFFICE
4--AUG-1902

**EUROPEAN AGENCY**, J. B. BAILLIERE & FILS, 19 Rue Hautefeuille, Paris.

*Subscription, if paid in advance, $2.00; otherwise, $2.50. Foreign subscription, $2.50*

Entered at the Post Office at Buffalo, N. Y., as second-class mail matter.

A. T. BROWN PRINTING HOUSE, BUFFALO, N. Y.

**Table of Contents on Page V.**

